# A site specific model and analysis of the neutral somatic mutation rate in whole-genome cancer data

**DOI:** 10.1186/s12859-018-2141-2

**Published:** 2018-04-19

**Authors:** Johanna Bertl, Qianyun Guo, Malene Juul, Søren Besenbacher, Morten Muhlig Nielsen, Henrik Hornshøj, Jakob Skou Pedersen, Asger Hobolth

**Affiliations:** 1 Department of Molecular Medicine, Aarhus University, Palle Juul-Jensens Boulevard 99, Aarhus N, DK-8200 Denmark; 2 Bioinformatics Research Centre, Aarhus University, C.F. Mollers Alle 8, Aarhus C, DK-8000 Denmark

**Keywords:** Multinomial logistic regression, Site-specific model, Somatic cancer mutations

## Abstract

**Background:**

Detailed modelling of the neutral mutational process in cancer cells is crucial for identifying driver mutations and understanding the mutational mechanisms that act during cancer development. The neutral mutational process is very complex: whole-genome analyses have revealed that the mutation rate differs between cancer types, between patients and along the genome depending on the genetic and epigenetic context. Therefore, methods that predict the number of different types of mutations in regions or specific genomic elements must consider local genomic explanatory variables. A major drawback of most methods is the need to average the explanatory variables across the entire region or genomic element. This procedure is particularly problematic if the explanatory variable varies dramatically in the element under consideration.

**Results:**

To take into account the fine scale of the explanatory variables, we model the probabilities of different types of mutations for each position in the genome by multinomial logistic regression. We analyse 505 cancer genomes from 14 different cancer types and compare the performance in predicting mutation rate for both regional based models and site-specific models. We show that for 1000 randomly selected genomic positions, the site-specific model predicts the mutation rate much better than regional based models.

We use a forward selection procedure to identify the most important explanatory variables. The procedure identifies site-specific conservation (phyloP), replication timing, and expression level as the best predictors for the mutation rate. Finally, our model confirms and quantifies certain well-known mutational signatures.

**Conclusion:**

We find that our site-specific multinomial regression model outperforms the regional based models. The possibility of including genomic variables on different scales and patient specific variables makes it a versatile framework for studying different mutational mechanisms. Our model can serve as the neutral null model for the mutational process; regions that deviate from the null model are candidates for elements that drive cancer development.

**Electronic supplementary material:**

The online version of this article (10.1186/s12859-018-2141-2) contains supplementary material, which is available to authorized users.

## Background

Cancer is driven by somatic mutations that convey a selective advantage to the cell. However, in most cases the somatic mutation rate in cancer cells is considerably higher than in healthy tissues, while only a small fraction of the mutations are thought to be associated with cancer development [1]. The majority of the mutations are neutral and are caused by perturbed cell division, maintenance and repair or over-expression of mutagenic proteins (e.g. the APOBEC gene family [2]). A comprehensive framework of the random mutation process in cancer cells is key to identify the regions, pathways and functional units that are under positive selection during cancer development.

The mutation rate varies along the genome, depending on genomic properties of the position such as the sequence context (e.g. the 5’ and 3’ nucleotides; [3]), chromatin organisation [4] or replication timing [5]. Many studies have investigated what determines the mutation rate and what kind of models should be used [5] modeled the mutation heterogeneity using local regression with expression level and replication timing as explanatory variables [4] applied random forest regression on mutation counts in 1Mb windows using histone modifications and the density of DNase I hypersensitive sites. [6] predicted the number of mutations per element by a beta-binomial distribution using replication timing and noncoding annotations such as promoter, UTR and ultra-conserved sites. Unlike these approaches that segmented the genome into regions according to the explanatory variables and estimated separate models for them, in a site-specific regression model, this division is not necessary [7] implemented a Poisson-binomial model on 50kb windows, where they used logistic regression to predict the position-specific mutation probability, based on base-pair, replication timing and the presence and type of transcript. Here, we propose a framework based on multinomial regression that is able to model different types of substitutions. Unlike region-based models, we can use site-specific explanatory variables without dividing the data into subsets. In this way, we use the full dataset to estimate the regression coefficients for all the explanatory variables. We include the trinucleotide context, GC content and CpG island annotations to describe the local base composition. Local properties of the genome related to transcription and replication are taken into account using expression level, replication timing, DNase I hypersensitivity and genomic element types. To study the differences between somatic mutations and germline substitutions, we include the conservation score phyloP. We also include an explanatory variable to mask the repeat regions in the genome as mutation calls at repeat regions are biased due to technical reasons.

Generalized linear models also provide an interpretable framework for modelling and hypothesis testing. For example, we can estimate the mutation rates of CpG sites within and outside CpG islands, and test if they are different, and a patient-specific intercept allows us to take the large variation of mutation rates between patients into account.

**Fig. 1:**
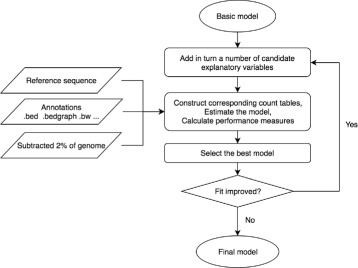
Workflow of the forward model selection procedure. The forward model selection is implemented on 2% of the data to determine the explanatory variables included in the final model. In each iteration of the model selection procedure, data tables are generated to summarize the site-specific annotations. The performance of the models is measured with the deviance loss obtained by cross-validation. The explanatory variable with the best performance is included in the set of variables for the next iteration. Parameter estimation for the final model is based on the remaining 98% of the data

Here, we analyse 505 cancer genomes from 14 different cancer types [8]. We compare the performance in predicting mutation probabilities of region-based Poisson models, site-specific binomial models and site-specific multinomial models. The site-specific multinomial model can predict both the overall mutation rate and mutation types accurately. We use a forward model selection procedure to compare and identify the explanatory variables that best explain the heterogeneity of the site-specific mutational process (Fig. 1).

The forward model selection procedure is implemented using 2% of the data while the final model fit is obtained from the remaining 98%. We find that site-specific conservation (phyloP), replication timing and expression level are the best predictors for the mutation rate. In general, the framework allows formal testing for inclusion of explanatory variables and also interaction terms. The impact of different explanatory variables can be inferred from the parameter estimates of our final model as the multiplicative changes in mutation rate. Our analysis confirms some known mutational signatures and it identifies associations with genomic variables like replication timing. It can also be used as the null model for cancer driver detection [9] and other applications that rely on a model of the mutation rate (e.g. identification of the tissue of origin for tumors of unknown primary, [4]).

## Results

### Heterogeneity of the mutation rate

**Fig. 2:**
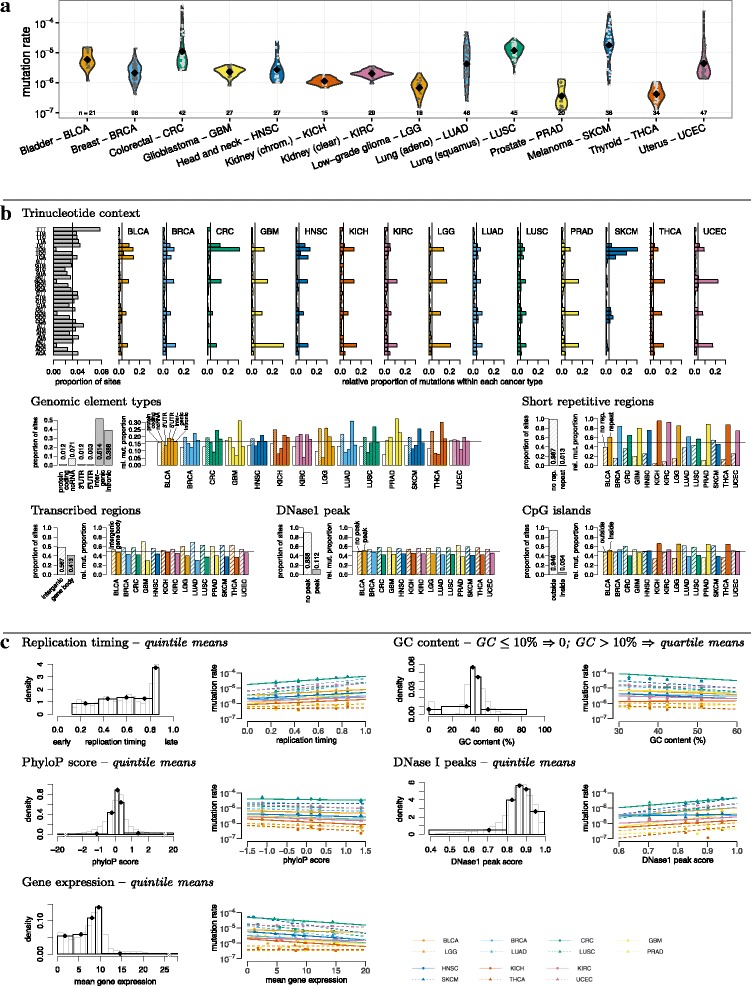
Heterogeneity of the mutation rate and explanatory variables. **a** Heterogeneity among cancer types and samples. Violin plot for the mutation probability for 14 cancer types. **b** Heterogeneity along the genome and the correlation with categorical explanatory variables. Relative proportion of mutations from nucleotide C or T in the neighboring context A,G,C,T (2·4·4=32 possibilities), relative proportion of mutations of six different genomic elements, and relative proportion of mutations within and outside repeat regions or CpG islands. **c** Heterogeneity correlated with continuous variables. Left column: continuous variables. Middle column: The continuous annotations are discretized into bins according to quantiles for site-specific regression models. Each bin is represented by the mean value within the bin. Grey transparent histograms: distribution of the continuous values of the annotation along the genome. Black transparent histograms: distribution of the discrete bins of the annotation (binning scheme in italics in the column “Annotation”). Black diamonds: Discrete value used for the binning. Right column: Predicted (lines) and observed (points) mutation rate for each cancer type and explanatory variables. The regression lines are generated under a multinomial logistic regression model using only the corresponding explanatory variable. Details about the different data types can be found in “Somatic mutation dataset” section

We observe significant heterogeneities of the mutation rate at multiple levels (Fig. 2). The mutation rate varies among cancer types (Fig. 2a): skin cutaneous melanoma, colorectal cancer and lung adenocarcinoma are the cancer types with the highest mean mutation rates (5–10 mut/patient/Mb) while thyroid carcinoma, prostate adenocarcinoma and low-grade glioma have the lowest (0.5–1 mut/patient/Mb). The mutation rate also differs between samples from the same cancer type, with the largest variation seen for skin cutaneous melanoma (the mutation rate ranges from 1 mut/patient/Mb to 150 mut/patient/Mb).

The mutation rate also varies between different genomic contexts (Fig. 2b). As previously shown, we find that mutational signatures are cancer type specific by looking into the mutation rate for different trinucleotide contexts. For instance, the mutation rate at TC* sites is particularly high in skin cutaneous melanoma, with the largest proportion of mutations at TCC positions of all cancer types. The mutation rate at CpG sites is elevated in all the cancer types. In colorectal cancer, we observe a high proportion of mutations at TCG and TCT sites. These can be attributed to mutations in the *POLE* gene that cause DNA polymerase *ε* deficiency [10]: we find an increased overall mutation rate, a very high proportion of T[C >A]T and T[C >T]G mutations and a high contribution of COSMIC signature 10 in six out of 42 colon cancer samples [11]. Five of those (and three of the other colon cancer samples) have a nonsynonymous mutation in *POLE* and one of them in addition in *POLD1*, which encodes the DNA polymerase *δ* (Additional file 1: Figure S1). When the samples with *POLE* mutation pattern are removed, the mutation pattern of the remaining colon cancer samples is very similar to most cancer types with a high proportion of mutations at CpG positions (Additional file 1: Figure S2).

The mutation rate also differes between genomic environments defined by the explanatory variables (Fig. 2b, c). Coding regions tend to have fewer mutations in all cancer types. Mutation rates are elevated for simple repeat regions, which might be related to mapping artefacts and ensuing technical challenges during mutation calling. The effect of CpG islands varies between different cancer types. The mutation rate in CpG islands is higher than in regions outside for thyroid carcinoma, prostate adenocarcinoma, low-grade glioma and kidney chromophobe, while for colorectal cancer, lung adenocarcinoma, lung squamous cell carcinoma and skin cutaneous melanoma the situation is reversed. Regions that are late replicated, GC rich, evolutionarily less conserved, inside DNase 1 peaks and lowly expressed have an elevated mutation rate. The explanatory power of the variables varies across cancer types as shown by the regression lines in Fig. 2c.

### Granularity of regression models

**Fig. 3:**
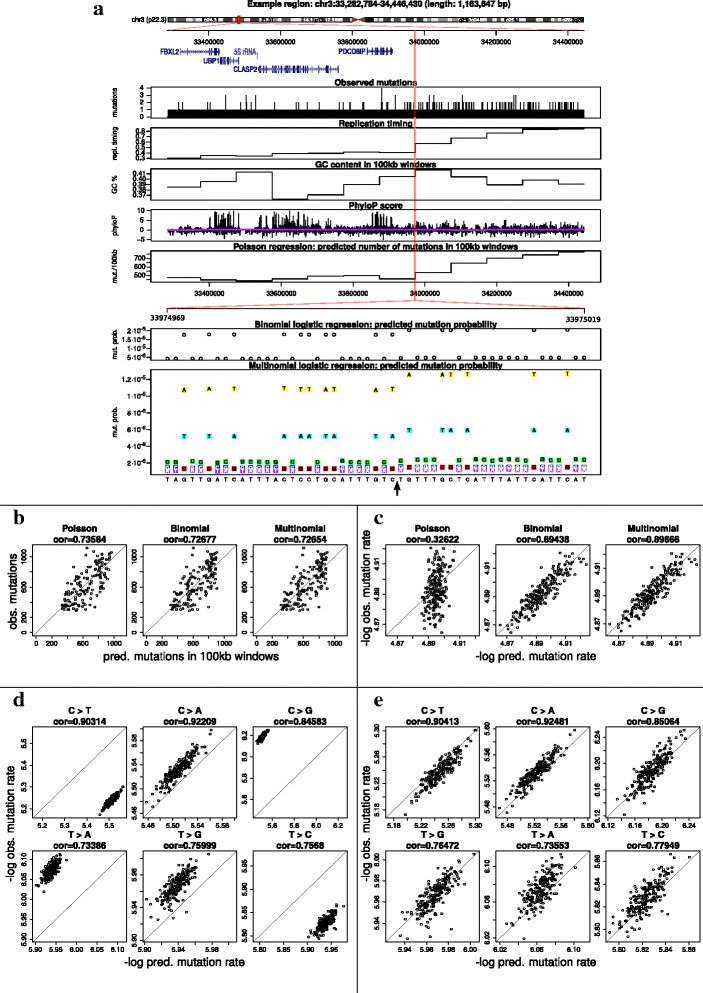
Comparison of Poisson regression model, site-specific binomial logistic regression model and site-specific multinomial logistic regression model. **a** Motivation (site-specificity) and conceptual explanation of the different models. Consider a 1.2 Mb region on Chromosome 3. We observe a number of mutations and the value of the explanatory variables replication timing, GC content and phyloP score. Given the values of the explanatory variables we use Poisson, site-specific binomial logistic regression or site-specific multinomial logistic regression to predict the number of mutations in a region (Poisson), the probability of a mutation in a single site (binomial) or even the probability of the three types of mutation in a single site (multinomial). **b** Predicted versus observed number of mutations for the three models for 100 kb regions. **c** Site-specific models perform substantially better in 1000 randomly selected sites. **d** The prediction for different mutation types with binomial logistic regression model in 1000 randomly selected sites. **e** The prediction for different mutation types with multinomial logistic regression model in 1000 randomly selected sites

We model the mutation probability in cancer genomes using a set of regression models. The most coarse-grained description of the number of mutations in a region is a Poisson regression count model and the most fine-grained is a binomial or multinomial site-specific regression model. Here we describe and investigate in detail the (dis)advantages of these three models. A conceptual overview of the models is given in Fig. 3a.

#### Poisson count regression model

In Poisson regression, the number of mutations in a genomic region of fixed length is modeled. The whole genome is divided into regions of pre-fixed length or according to the value of explanatory variables (e.g. segmented by genomic element types). Regression modelling is facilitated by summing the mutation counts and summarizing the annotations over the region.

We model the mutation count *N*_*r*,*sam*_ in the *r*-th genomic region with length *L*_*r*_ in sample *sam*. Furthermore, *can* is the cancer type of the sample. Mutations arise randomly with probability *p*_*r*,*sam*_. As *p*_*r*,*sam*_ is small and *L*_*r*_ is large, we have the Poisson approximation of the binomially distributed number of mutations

$$N_{r, sam} \sim \text{Bi}(L_{r}, p_{r, sam}) \approx \text{Po}(L_{r} p_{r, sam}). $$

For *J* explanatory variables, the expected mutation count *λ*_*r*,*sam*_=*L*_*r*_*p*_*r*,*sam*_ can be modeled by Poisson regression with a log-link:

$$\log \lambda_{r, sam} = \mu_{sam} + \beta_{1, can} x_{r, 1} + \dots + \beta_{J, can} x_{r,J} $$

where for the *j*th explanatory variable, *x*_*r*,*j*_ is the average value of the annotation across region *r* if the explanatory variable is continuous and for categorical explanatory variables, *x*_*r*,*j*_ is derived from the proportions of different levels of the annotations in region *r*, *j*=1,…,*J*. *μ*_*sam*_ is the sample-specific intercept.

#### Site-specific binomial regression model

In site-specific regression models, the mutation probability is modeled in each position of the genome. We enable regression modelling by binning the continuous annotations, such that we are able to sum mutation counts over positions with the same combination of annotations, and thereby reduce the size of the data set. We consider both site-specific binomial and multinomial regression models.

We model the mutation probability *p*_*i*,*sam*_ at a site *i* in sample *sam* of cancer type *can*. With a logit link, the mutation probability can be modeled by logistic regression:

$$ \log \frac{p_{i, sam}}{1 - p_{i,sam}} = \mu_{sam} + \beta_{1,can}x_{i,1} + \dots + \beta_{J, can}x_{i,J} $$

where *x*_*i*,*j*_ is the value of the *j*th explanatory variable at site *i*.

#### Site-specific multinomial regression model for strand-symmetric mutation types

We model the mutation probability for different mutation types. Assuming strand-symmetry, we are not distinguishing between e.g. A >G (A to T) mutations and T >C mutations, *p*^*A*>*G*^=*p*^*T*>*C*^. We consider the strand with the C or T nucleotide, and the mutation probability matrix is

$$\begin{aligned} & \qquad\mathbf{A} \quad\qquad \mathbf{G} \quad\qquad \mathbf{C} \quad\qquad \mathbf{T} \\ \begin{array}{c} \mathbf{A}~\\ \mathbf{G}~\\ \mathbf{C}~\\ \mathbf{T}~\\ \end{array} &\left(\begin{array}{cccc} p^{{T > T}} & \quad p^{{T > C}} & \quad p^{{T > G}} & \quad p^{{T > A}} \\ p^{{C > T}} & \quad p^{{C > C}} & \quad p^{{C > G}} & \quad p^{{C > A}} \\ p^{{C > A}} & \quad p^{{C > G}} & \quad p^{{C > C}} & \quad p^{{C > T}} \\ p^{{T > A}} & \quad p^{{T > G}} & \quad p^{{T > C}} & \quad p^{{T > T}} \\ \end{array}\right) \end{aligned} $$

with only 6 types of mutations.

We model these mutation probabilities by setting up a multinomial logistic regression model, where dummy variables are used to distinguish between mutations from (G:C) base pairs and mutations from (A:T) base pairs. The (G:C) basepairs are modelled with probabilities

$$\left(p_{i,\text{sam}}^{{C > A}}, p_{i,\text{sam}}^{{C > G}},p_{i,\text{sam}}^{{C > C}}, p_{i,\text{sam}}^{{C > T}} \right)$$

for (G:C) position *i* in sample *sam*. With *J* explanatory variables, the mutation probability at G:C position *i* in sample *sam* of cancer type *can* can be written as:

$$\begin{array}{*{20}l} \log \frac{p_{i,\text{sam}}^{{C > A}}}{p_{i,\text{sam}}^{{C > C}}} =\mu_{\text{sam}}^{{C > A}} + \beta_{1,\text{can}}^{{C > A}} x_{i,1} + \cdots + \beta_{k,\text{can}}^{{C > A}} x_{i,J} \\ \log \frac{p_{i,\text{sam}}^{{C > G}}}{p_{i,\text{sam}}^{{C > C}}} = \mu_{\text{sam}}^{{C > G}} + \beta_{1,\text{can}}^{{C > G}} x_{i,1} + \cdots + \beta_{k,\text{can}}^{{C > G}} x_{i,J} \\ \log \frac{p_{i,\text{sam}}^{{C > T}}}{p_{i,\text{sam}}^{{C > C}}} = \mu_{\text{sam}}^{{C > T}} + \beta_{1,\text{can}}^{{C > T}} x_{i,1} + \cdots + \beta_{k,\text{can}}^{{C > T}} x_{i,J}. \end{array} $$

Note that the probability for no mutation $p_{i, sam}^{{C > C}}$ is the reference. Similarly, (A:T) basepairs are modeled with probabilities

$$\left(p_{i,\text{sam}}^{{T > A}}, p_{i,\text{sam}}^{{T > G}},p_{i,\text{sam}}^{{T > C}}, p_{i,\text{sam}}^{{T > T}} \right)$$

and the reference is the probability for no mutation $p_{i, sam}^{{T > T}}$. We compare the performance of the three models on 2% of the whole genome data.

The setting for the three models is shown in Fig. 3a. Since we are mainly interested in their predictive performance, we have not included overdispersed models. Overdispersion is a way to model unexplained variance in the data, but it does not qualitatively change the predictions of a model. Each model is trained with replication timing, phyloP, and context information from the reference genome. For the region-based Poisson model, continuous annotation values are averaged over the selected region and GC percentages are calculated for each region. For the site-specific models, we use the site-specific annotations for each site. Continuous values are discretized to simplify the estimation process.

The results are shown in Fig. 3b-e. We compare the performance of these models at different resolutions using different datasets. In Fig. 3b, we predict the mutation counts in large windows of length 100kb. In Fig. 3c, d, e, we predict the mutation counts in sets of 1000 randomly sampled individual sites. When predicting mutation counts in large windows (100 kb), the three regression models perform similarly. For prediction in a randomly selected small number of sites (1 kb), the two site-specific models out-perform the region-based model (Fig. 3c). The site-specific models can capture the mutational heterogeneities between sites and provide a more accurate mutation probability at any resolution. This is in contrast to the region-based model where a large number of sites are required for accurate predictions. In addition to predicting the probability for mutation events, the multinomial regression model can also predict the probabilities of different mutation types. It outperforms the binomial model by taking the different mutation rates into account (Fig. 3d, e).

### Model selection

We consider the site-specific multinomial regression model to predict mutation probabilities for different mutation types at a single site. We implement a forward model selection procedure to determine the explanatory variables in the final model (Fig. 1). In each step, we add all possible new variables to the previous model in turn and rank the resulting new models. We identify and include the explanatory variable with the best fit and iterate the procedure several times. With forward model selection, we avoid the preparation of large analytical data tables that contain all potential explanatory variables (“Preparation of the analytical data table” section). We choose the deviance loss that measures the predictive performance of the model to assess the fit. By estimating it by cross-validation, we avoid overfitting, because it assesses the fit on an independent subset of the data (“Cross validation” section).

**Fig. 4:**
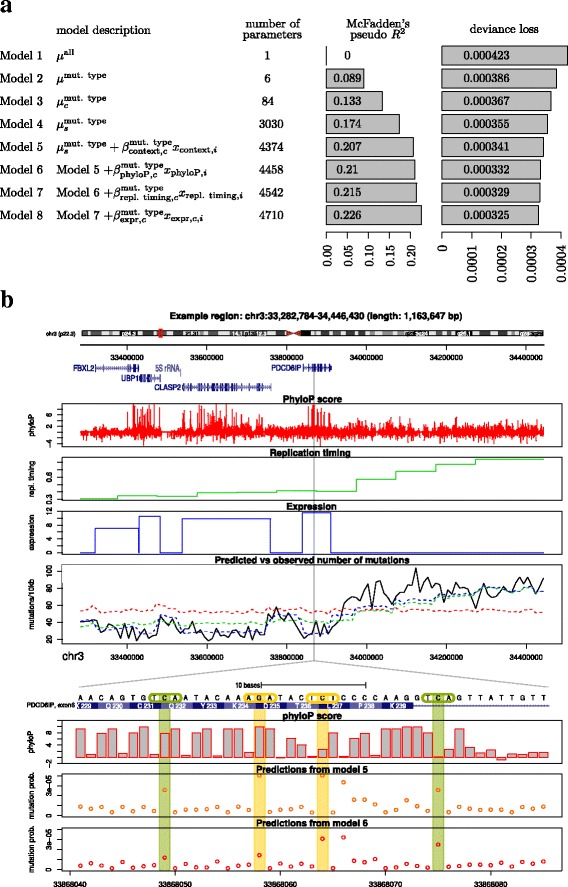
Model selection results. **a** Improvement of the fit during forward model selection. In each iteration, we estimate the deviance loss by cross validation to determine which explanatory variable to include in the next model. **b** Explanatory variables and predicted vs observed number of mutations along the genome in an example region on chromosome 3 for models 6–8. Zoom: DNA sequence, phyloP score and predicted mutation probabilities from models 5 and 6

By construction, the fit improves during the model selection procedure (Fig. 4a). We also evaluate McFadden’s pseudo *R*^2^ as a measure of the explained variance that is valid in categorical regression models (“McFadden’s pseudo *R*^2^” section).

As a reference model, we start out with a single mutation rate for the whole genome in all samples (Model 1; Fig. 4a). This model cannot explain any of the variation in the mutation rate between samples and positions, so McFadden’s pseudo *R*^2^=0. After including the six strand-symmetric mutation types in the model (Model 2), we add cancer and sample specific intercepts (Model 3 and Model 4) to make sure that we account for sample-specific mutation rates. In the next step, we include the left and right neighboring base-pair for each cancer type (Model 5).

Starting with Model 5, additional annotations are added using forward model selection. We consider the phyloP score, replication timing, expression, genomic segments, GC content in 1 kb, CpG islands, simple repeats and DNase I hypersensitivity. For each of these variables, cancer specific regression coefficients are estimated to allow for differences in the mutational process between cancer types. For expression, we use data directly obtained from matching tumor types, so we also take expression differences between cancer types into account.

The annotation with the largest decrease in the deviance loss function is the phyloP score (Model 6). Subsequently, replication timing (Model 7) and gene expression (Model 8) are added. At this point, we stop the model selection procedure to avoid the time-consuming creation of larger count tables (see “Preparation of the analytical data table” section for details) as we also see that the improvement of the fit levels off. Detailed results for each step of the forward selection procedure are provided in Additional file 1: Section S2.1. To assess the robustness of the results, we rerun the forward model selection procedure five times on randomly selected regions that cover 2% of the whole genome. The ranking of the variables is constant for all the experiments (Additional file 1: Section S2.2).

Adding the context in model 5 gives a substantial improvement over model 4. When the phyloP score is added in model 6, it can be seen in Fig. 4b that it considerably changes the predicted mutation rate for the same nucleotide triplet at two positions with different phyloP score. While the trinucleotide context and the phyloP score vary on a basepair scale, both replication timing and expression vary on a kilo-base scale. Even though much of the per-base-pair variation in the mutation rate is already captured in Models 5 and 6, the long-range variation is considerably better explained in Model 7 and Model 8 (Fig. 4b). We can see that adding replication timing in Model 7 considerably changes the predicted mutation rate obtained from Model 6 that is relatively uniform in the 1.1 Mb region shown in the figure, by taking the replication timing gradient in the region into account. Finally, in Model 8, the mutation rate in highly expressed regions is lowered, which again improves the prediction.

Driver detection methods, such as MutSigCV [5] and ncdDetect [9], are generally based on a model of the neutral mutation rate in tumors. We use two typical cancer genes, the oncogene *KRAS* and the tumor suppressor gene *TP53*, to contrast the observed mutation pattern in a driver to the predicted neutral mutation rate.

In Additional file 1: Figure S4, it is obvious that the predicted mutation rate is lower at the gene body of *KRAS* than right outside of it. A zoom onto exon 2 shows a cluster of mutations at positions 25,398,281-5, with 25 mutations at position 25,398,284-5 which cause a change of protein. This cluster is highly unlikely under our neutral model.

For *TP53*, we also find a lower predicted mutation rate at the gene body of *TP53* and the overlapping gene *WRAP53* (Additional file 1: Figure S5). Here, the observed mutations are more spread than in KRAS, but they mainly occur in highly conserved exonic regions, where the neutral model predicts a low mutation rate.

### Estimation results

Upon determining the final model from the model selection procedure, we estimate parameters for the multinomial logistic regression model on the remaining 98% of the genome. To study the difference between cancer types, all position-specific explanatory variables are stratified by cancer type. The coefficients represent multiplicative changes in mutation rate. Our results confirm the large differences in mutation pattern both between samples and cancer types, but also between different genomic and epigenomic regions.

**Fig. 5:**
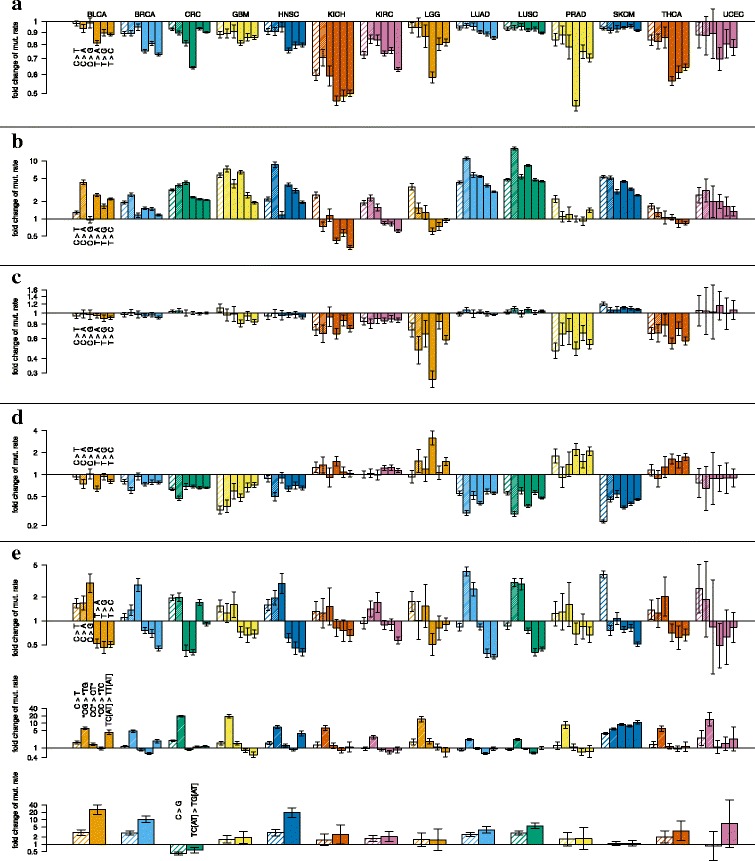
Parameter estimation results. **a** Neutral vs. conserved regions. The height of the bars give the fold change in mutation rate in conserved regions (phyloP score =1.3838, mean of the highest quintile, see Fig. 2c compared to neutral regions (phyloP score = 0). **b** Early vs. late replicating regions. The height of the bars give the fold increase/decrease in mutation rate in late replicating regions (replication timing = 1) compared to early replicating regions (replication timing = 0). **c** Intergenic vs. gene body. The height of the bars give the fold increase/decrease in mutation rate in gene bodies compared to intergenic regions. **d** Low vs. high expression. The height of the bars give the fold increase/decrease in mutation rate in highly expression regions (expression value =15, approx. the mean of the highest quintile, see Fig. 2c compared to lowly expressed regions (expression value = 0). Here, only gene bodies are considered. **e** Mutation type are considered as the combination of substitutions and neighboring sites. The horizontal line indicates the average mutation rate for each cancer type. The height of the bars give the fold increase/decrease in mutation rate for a specific mutation type. Panel 1: Different substitution types. Panel 2: C >T mutations in different contexts. Panel 3: C >G mutations in all contexts and in TpCp[AT] contexts

We observe that regions that have been highly conserved during human evolution have a lower mutation rate in all cancer types and for all mutation types, and this difference is nearly always significant (Fig. 5a). For kidney chromophobe and prostate adenocarcinoma, it is reduced to less than half for some of the mutation types. In breast cancer, head and neck squamous cell carcinoma, kidney chromophobe and thyroid carcinoma, this difference is much more pronounced at A:T positions than at G:C positions, but there is no general pattern with respect to the mutation type.

As previously described [5], we nearly always find a positive association between replication timing and mutation rates (see regression lines in Fig. 2c; later replicating regions have more mutations), but the regression coefficient varies significantly between the different cancer types and the mutation types (Fig. 5b). For the dummy variable that distinguishes gene bodies, where expression is measured, from intergenic regions, we see a mixed pattern of insignificant and negative regression coefficients where the mutation rate in gene bodies is reduced to up to one third of the rate in intergenic regions (low-grade glioma; Fig. 5c). The only exception is melanoma with a slightly increased mutation rate in gene bodies.

Also when we consider regions within gene bodies, we find a reduced mutation probability in highly expressed regions for most cancer types. The most extreme example is the probability of a C > T mutation in highly expressed regions in melanoma, which is only one fifth of the probability in lowly expressed ones (Fig. 5d). Low-grade glioma, prostate adenocarcinoma and thyroid cancer show the opposite pattern for some of the mutation types, though. However, this effect is dampened by the dummy variables for the gene body that have comparably large coefficients of the opposite sign for these three cancer types.

We find that the mutation rates differ between the different types of mutations, but also between the specific contexts that we consider: CpG, to capture the pattern of spontaneous deamination, and TpCp[AT], to capture the APOBEC signature. We find that the C > T mutation rate is higher in CpG sites than in other sites in all cancer types. In skin cutaneous melanoma, we also observe elevated mutation rate for CC context, which is related to the elevated CC > TT mutation rate due to UV light [12]. We observe elevated rates of mutations that fit the APOBEC pattern in breast cancer, bladder urothelial carcinoma, head and neck squamous cell carcinoma, lung squamous cell carcinoma and skin cutaneous melanoma.

## Discussion and conclusions

We use a multinomial logistic regression model to analyse the somatic mutation rate in each position of a cancer genome. We consider various genomic features, such as the local base composition, the functional impact of a region and replication timing. Because of the site-specific formulation, the model is the most fine-grained description of the mutation rate, while it can still take genomic properties into account that vary or are measured on a longer scale, like replication timing and expression levels.

The mutational spectrum and intensity are known to vary considerably between cancer types as well as between patients with the same cancer type [5]. In our analyses, we capture a considerable part of the variance by including cancer type and sample specific mutation rates.

We find the site-specific phyloP conservation scores to explain more than any of the other explanatory variables we tested. The conservation scores reflect the germline substitution rate between species through evolution and are designed to capture the effect of selection [13]. However, somatic evolution of the cancer genome is not subject to the same constraints as germline evolution. In particular, purifying selection may be relaxed as many genes and regulatory elements that are needed during the organismal life cycle are dispensable for the growth of cancer cells [14]. Instead, the conservation scores’ explanatory power in our study may stem from their correlation with genes and other functional elements, with special properties that affect their mutation rate. In particular, it is known that the mutation rate is elevated at transcription factor binding sites in some cancer types [15]. Furthermore, the phyloP scores are also correlated with expression regions and open chromatin, which both have decreased mutation rates due to transcription-coupled repair and other repair mechanisms [16].

The second explanatory variable added is replication timing. This has not only been found for cancer genomes [5], but also for germline mutations [17] and somatic mutations in healthy tissue [18]. In late replicating regions, single stranded DNA (ssDNA), which is susceptible to mutagenic processes like deamination, accumulates [17]. Varying intensities of such mutational processes could explain the differences in the impact of replication timing between the cancer types and the mutation types. The different strengths of the associations between replication timing and the mutation rate can also be attributed to differences in replication timing between cell types [19] that may be missed in the replication timing dataset at use, which is obtained from HeLa cell lines [20].

Only after the phyloP score and replication timing, the cancer type specific expression level is added to the model. This can be explained by the fact that the phyloP score and expression are correlated, as mentioned above, and so are replication timing and expression [21]: protein-coding genes are concentrated in early replicating regions. However, the expression levels that we use are tissue specific and might therefore improve the fit of the model further.

By separately estimating a regression coefficient for the gene-body and one for the expression level within gene bodies, we can potentially distinguish the general properties of genes, like location with respect to replication timing origins and sequence composition, and the intensity of transcription-coupled repair (TCR). Since TCR is a subpathway of nucleotide excision repair (NER), it is expected to act on helix-distorting mutations like for example the well-known UV light induced CC > TT mutations in melanoma. Thus, the cancer specific differences that we see might be explained by varying effectiveness of TCR, but also by different proportions of mutations that create bulky distortions. For example, the rate of C > T mutations is further decreased in highly expressed regions in melanoma than the other mutation types.

The parameters can be interpreted as multiplicative mutation rate changes. By using interaction terms, it is straightforward to analyse and test differences in mutation rate between cancer types, samples or specific genomic regions of interest. Furthermore, generalized linear models come with a natural framework for hypothesis testing, e.g. to compare two subcohorts of interest within the same model, like *POLE* mutated versus *POLE* wildtype colorectal cancer samples, or NER deficient tumors versus NER proficient ones. It would be interesting to compare our model-based description of the mutation rate to unsupervised learning of mutational signatures by matrix factorization [3].

In the logistic regression model, we can estimate cancer type specific regression coefficients to capture the differences between cancer types. We can also include tissue specific explanatory variables if there are measurements for the corresponding cancer tissue or matching healthy tissue available. This is of particular interest for epigentic measurements like histone modifications, where the tissue of origin has been shown to be informative [4].

Patient-specific characteristics can also be added as explanatory variables. The age of the patients could be used to study clock-like mutational processes [11]. Known somatic or germline mutations that are associated with specific mutational processes or repair pathways can also be used as explanatory variables, e. g. a germline deletion of *APOBEC3B* that fuses APOBEC3A with the 3’ UTR of APOBEC3B has been found to be associated with an increased number of APOBEC-type mutations [22]. The impact of this mutation could be studied by including an interaction term with the TpCp[AT] positions.

Our model is very flexible and versatile and the explanatory variables can be customized according to different applications. We provide software that facilitates similar position-specific analyses on new mutation datasets with a user-specified set of genomic annotations as explanatory variables. First, an analytical data table is created for the whole genome or a specified set of genomic regions (implemented in python). This table can be updated with new samples at any time. Then, a multinomial logistic model can be estimated. This is implemented in R and mimics the interface of standard R functions like glm.

An important application of the model is the prediction of the somatic mutation probability under the assumption of neutrality. In our driver detection method ncdDetect [9], we use the position-specific predictions that we obtain from the multinomial model to evaluate if the mutation rates or their distributions are significantly different from the expectation under neutrality. This allows a flexible analysis of regions of any size. Even non-contiguous regions with very different properties than the overall genomic patterns can be investigated.

## Methods

### Data

#### Somatic mutation dataset

We use SNV calls from 505 tumor-normal samples across 14 different cancer types [8]. We build our data set based on the UCSC hg19 assembly. We removed regions with low mappability, ultra-high mutation rates and lacking annotation. Problematic regions for NGS alignments identified for the ENCODE project [23] were subtracted. Low mappability regions, defined by the GEM tool [24] and CRG Alignability track from UCSC with mappability less than 0.5 in 100-mers, were also subtracted. Hyper-mutated genomic segments containing Immunoglobulin/T-cell receptor (IG/TR) genes defined by GENCODE together with 10 kb flanking regions, combined when less than 100 kb in distance, were excluded from analysis. We also excluded sites on ChrX and ChrY, because for some of the annotation files we lack information for one or both of the sex chromosomes.

A total number of 14,200,393 SNVs are observed in the subtracted regions for 505 samples across 14 cancer types.

##### Genomic element types

We divided the genome into six types of genomic elements: coding, 5’ UTR, 3’UTR, ncRNA, intron and intergenic. Based on the GENCODE v.19 transcripts, coding regions, 5’ UTR regions and 3’ UTR regions as well as introns were defined for protein-coding transcripts. Non-coding RNA regions were defined as all remaining regions in the full transcript set. All remaining bases were categorized as intergenic.

##### GC content

We calculate the percentage of G:C base pairs in 1 kb windows based on the reference genome. Regions with GC percentage less than 10% are annotated with value 0. Other regions are discretized into quartiles.

##### CpG islands

We segmented the genome by presence or absence of CpG islands. The CGI Mountain annotation from CgiHunter (http://cgihunter.bioinf.mpi-inf.mpg.de/) was used. The CGI Mountain score quantifies if a region is a CpG island. Scores above zero indicate a CpG island. We use a dummy variable derived from the CGI Mountain score in our analysis indicating whether the CGI Mountain score is larger than zero.

##### Simple repeats

We annotated the simple repeat regions in the genome according to RepeatMasker (http://www.repeatmasker.org), which defines the interspersed and low-complexity repeats in hg19. We use a dummy variable to indicate whether a genomic site is in a region masked as simple repeats.

##### DNase I peaks

We defined DNase I peaks according to the DNase I annotation from the Roadmap Epigenomics project [25]. We use the score from HoneyBadger2 to indicate the DNase I signal strength for regions with a DNase I peak (http://www.broadinstitute.org/~meuleman/reg2map/). The regions not annotated in the HoneyBadger2 were masked as “no peak” regions in our analysis. The values of peaks are approximatedly between 0.4 and 1, with 1 being highly accessible.

##### PhyloP score

The conservation score phyloP (phylogenetic *p*-values) is part of the PHAST package (http://compgen.bscb.cornell.edu/phast/). We used the score from the multiple alignments of 99 vertebrate genomes to the human genome [13]. We use the version of phyloP100way which covers 99.8*%* of the subtracted regions.

##### Replication timing

We adjust the replication timing data from [20] to the hg19 assembly. The replication timing values range from 0 to 1, indicating earlier to later replicating regions. The replication timing annotation covers 91.2*%* of the subtracted regions.

##### Gene expression

We define the gene expression level according to TCGA RNAseq expression data. Expression data was $\log _{2}(x+1)$ transformed. For each cancer type, the median expression was calculated for all genes. If multiple annotations of a gene exist, the longest annotation is used. For overlapping genes, the expression is a cumulative sum.

As in [8], we collapse colon (COAD) and rectal carcinoma (READ) to a joint cancer type CRC by averaging across the expression values.

#### Preparation of the analytical data table

In order to facilitate the model fitting procedure, we summarized the genomic data into count tables. In Poisson count models, each row in the count table represents a pre-defined region. For continuous explanatory variables, such as replication timing, the annotations are averaged over the region. For categorical explanatory variables, the annotations are transformed to the percentage for different levels of the variables, e.g, the binary explanatory variable indicating whether the site is a (G:C) base pair or not is transformed to GC content of the window. In site-specific regression models, we discretized continuous variables into bins according to quartiles or quintiles. Each row in the count table represents the counts of mutations under a certain combination of levels of all the explanatory variables. As we are summarizing the whole genome in the count table, we expect to see all the combinations of the levels for all the explanatory variables. Thus, the sizes of the count tables grow significantly when adding new explanatory variables. The generation of the count tables also takes much longer time with more explanatory variables. Because of the space and time consumption, a large count table including all the explanatory variables is computationally infeasible. We implemented the forward model selection procedure to avoid many huge count tables. For each iteration in the forward model selection procedure, we made new count tables only for the selected sets of explanatory variables from previous step and one new candidate explanatory variable. We then added the best candidate in the explanatory variable set and repeated for the next step. We used 2% of the whole genome in the model selection and made a final count table with the remaining 98% sites only for the explanatory variables that were determined from the model selection procedure. The generation of the count table for the final model takes 6000 CPU hours on our cluster (2.5 GHz CPUs).

### Multinomial regression model

#### Estimation

The observations in the regression model are indexed by the genomic position *i*(1≤*i*≤2.56·10^9^) for all positions on chr1 to chr22 after excluding problematic regions and the samples *sam*(1≤*sam*≤505), so the total number of observations is 1.3·10^12^. Starting from Model 4, sample specific intercepts for the six mutation types sum up to 6·505=3030 parameters. The regression coefficients for explanatory variables are indexed by the 14 cancer types. In Model 5 each cancer type has 4·4·6=96 parameters for the neighboring context and the mutation type (4 for the left neighboring site, 4 for the right neighboring site and 6 for the mutations), so the total number of parameters in this model is 6·505+14·96=4374. From Model 6 to Model 8 we use 1 parameter for each of the continuous explanatory variables phyloP, replication timing and expression level. We also add 1 dummy variable for expression level indicating whether the given site is potentially expressed or not. In Model 6 we have 4374+14·6=4458 parameters. In Model 7 we have 4458+14·6=4542 parameters. In the final model we have 6·505+14·96+14·(3+1)·6=4710 parameters in total. To reduce memory usage and computation time, the parameters are estimated in three separate binary logistic regression models, but the variance-covariance matrix of the parameters is estimated for the multinomial model [26]. We have implemented an R-package for the estimation [27] that is based on the function glm4 from the contributed package MatrixModels [28]. It also includes estimation of the variance-covariance matrix using the package Matrix for efficient handling of large and sparse matrices [29].

#### Dirichlet prior and pseudo counts

If a model with many explanatory variables and interaction terms among them is estimated (e.g. sampleID × neighbors × strong), it can easily occur that for a certain combination of levels of categorical variables, there have been no mutations of a certain type observed. This case is especially likely, if the sampleID is involved. This causes numerical problems in the maximum likelihood estimation [30].

To solve this problem while still obtaining a positive mutation probability, we add pseudo counts to the observed mutation counts. This is equivalent to specifying a Dirichlet prior for the multinomial model, so it leads to the same point estimates as the posterior mean would in a Dirichlet-Multinomial model [31].

To reduce the impact of the pseudo counts, we only add them to combinations of levels with no observed mutations and we do not use a uniform distribution, but let them be proportional to the observed mutation counts from all samples of the corresponding cancer type. The mutation counts from the sample and from the cancer type are equally weighted.

Let $\textbf {n}_{K, \text {sam}} = \left (n_{K, \text {sam}}^{C>A}, n_{K, \text {sam}}^{C>G}, n_{K, \text {sam}}^{C>C}, n_{K, \text {sam}}^{C>T}\right)$ be the number of each type of mutation for the combination of levels *K* in sample *sam* with at least one of the mutation counts being zero, and $\textbf {n}_{K,\text {can}} = \left (n_{K, \text {can}}^{C>A}, n_{K, \text {can}}^{C>G}, n_{K,\text {can}}^{C>C}, n_{K, \text {can}}^{C>T}\right)$ be the mutation counts for combination *K* for all samples of cancer type *can*. Let *N*_*K*,sam_ and *N*_*K*,can_ be the total count of mutations of category *K* for the sample and cancer type, respectively. Then, the new count vector $\tilde {\textbf {n}}_{K, \text {sam}}$ is defined as

$$\tilde{\textbf{n}}_{K, \text{sam}} = \frac{1}{2}\textbf{n}_{K, \text{sam}} + \frac{N_{K, \text{sam}}}{2 N_{K, \text{can}}} \textbf{n}_{K, \text{can}}. $$

Consequently, the number of mutations per sample is preserved. The new mutation counts can be non-integers, but this can be handled by our implementation.

#### McFadden’s pseudo *R*^2^

To assess the fit of a model, we report McFadden’s pseudo *R*^2^

$$R^{2}_{\text{McFadden}} = 1 - \frac{\log L_{M}}{\log L_{0}},$$

where *L*_*M*_ is the likelihood of the model under investigation and *L*_0_ is the likelihood of a model with no predictors [32]. To measure the improvement of a model in comparison with the binomial model where there is no distinction between the mutation types, we use the same mutation probability for each mutation type in the model without predictors.

### Forward variable selection

To speed up data preparation, forward variable selection is conducted on a subset of approximately 2% of the genome, which is constructed by randomly selecting 60,000 windows of size 1 kb.

The cancer types kidney chromophobe, low-grade glioma, prostate adenocarcinoma and thyroid carcinoma have very low mutation counts, so they are disregarded during variable selection. We use cross-validation for forward variable selection. The improvement of the fit along the model selection procedure is measured with the deviance loss.

Starting with Model 5,

$$ \text{logit} \left(p_{i, sam}^{\text{mut. type}}\right) = \mu^{\text{mut. type}}_{sam} + \beta_{\text{context}, can}^{\text{mut. type}} x_{\text{context}, i},$$

additional terms of the form

$$\beta_{j, can}^{\text{mut. type}} x_{j, i}$$

with explanatory variable *j*∈ {phyloP, replication timing, genomic segment, expression, GC content, DNase 1, simple repeats, CpG island}, are selected following the forward selection scheme.

To test the robustness of the selected model, the forward variable selection procedure is repeated five times.

#### Cross validation

An assessment of the fit of a model can be obtained by cross validation. Five-fold cross validation is used to select the annotation with the highest explanatory power. To this end, the 1 kb windows of the variable selection subset are divided randomly among 5 sets. In turn, 4 of them are joined as the training set, on which the multinomial model is estimated, and the remaining set is used as the validation set to estimate the loss function.

The deviance loss function for the observed site *i* in sample *sam* is defined as

$$D_{i,sam} = -2 \sum_{k} \mathbbm{1}(y_{i, s}=k) \log \hat{p}(y_{i, s}=k)$$

where *k* denotes all possible mutation events at site *i*, *y*_*i*,*sam*_ the observed event at site *i* in sample *sam* and $\hat {p}(y_{i, sam}=k)$ the probability of event *k* estimated under the multinomial regression model [33]. Thus, it measures the prediction accuracy of the multinomial regression model. The total deviance loss of a model is

$$D = \sum_{i=1}^{N} \sum_{sam=1}^{505} D_{i, sam}$$

with *N* being the number of genomic positions.

## Additional file

Additional file 1 **Section S1***POLE* mutation patterns in colon cancer samples. **Section S2** Detailed forward model selection results. **Section S3** Mutation patterns in two cancer genes. (PDF 2222 kb)

## Abbreviations

NER: Nucleotide excision repair
TCR: Transcription-coupled repair
