# Supplementary material for: A site specific model and analysis of the neutral somatic mutation rate in whole-genome cancer data
Source: BMC Bioinformatics. 2018 Apr 19;19:147. doi: 10.1186/s12859-018-2141-2 (PMC5909259; doi:10.1186/s12859-018-2141-2)
Supplement: Supplementary file 1 — Section S1POLE mutation patterns in colon cancer samples. Section S2 Detailed forward model selection results. Section S3 Mutation patterns in two cancer genes. (PDF 2222 kb) [file 12859_2018_2141_MOESM1_ESM.pdf]

Supplementary Information

Johanna Bertl, Qianyun Guo, Malene Juul, Søren Besenbacher, Morten Muhlig Nielsen, Henrik Hornshøj, Jakob Skou Pedersen, Asger Hobolth

Contents

S1 *POLE* mutation patterns in colon cancer samples **1**

S2 Detailed forward model selection results **4**
S2.1 Loss estimates for all models under consideration 4
S2.2 Robustness of the model selection procedure 6

S3 Mutation patterns in two cancer genes **7**
S3.1 The oncogene *KRAS* 7
S3.2 The tumor suppressor gene *TP53* 8

S1 *POLE* mutation patterns in colon cancer samples

To identify polymerase epsilon deficient colon cancer samples, we first annotated nonsynonymous SNVs in the *POLE* and *POLD1* genes using FunSeq2 [Fu et al., 2014]. Second, we extracted the 30 mutational signatures (Alexandrov et al., 2015, downloaded from `http://cancer.sanger.ac.uk/cosmic/signatures`, Nov. 30, 2017) from each sample by non-negative least squares using the R-package `MutationalPatters` [Blokzijl et al., 2017] to identify samples with a particularly high number of signature 10 mutations [Alexandrov et al., 2013] (Fig. S1).

In Fig. S2, we show the proportion of mutations in each nucleotide triplet in colon cancer, stratified by the presence of *POLE* mutation patterns.

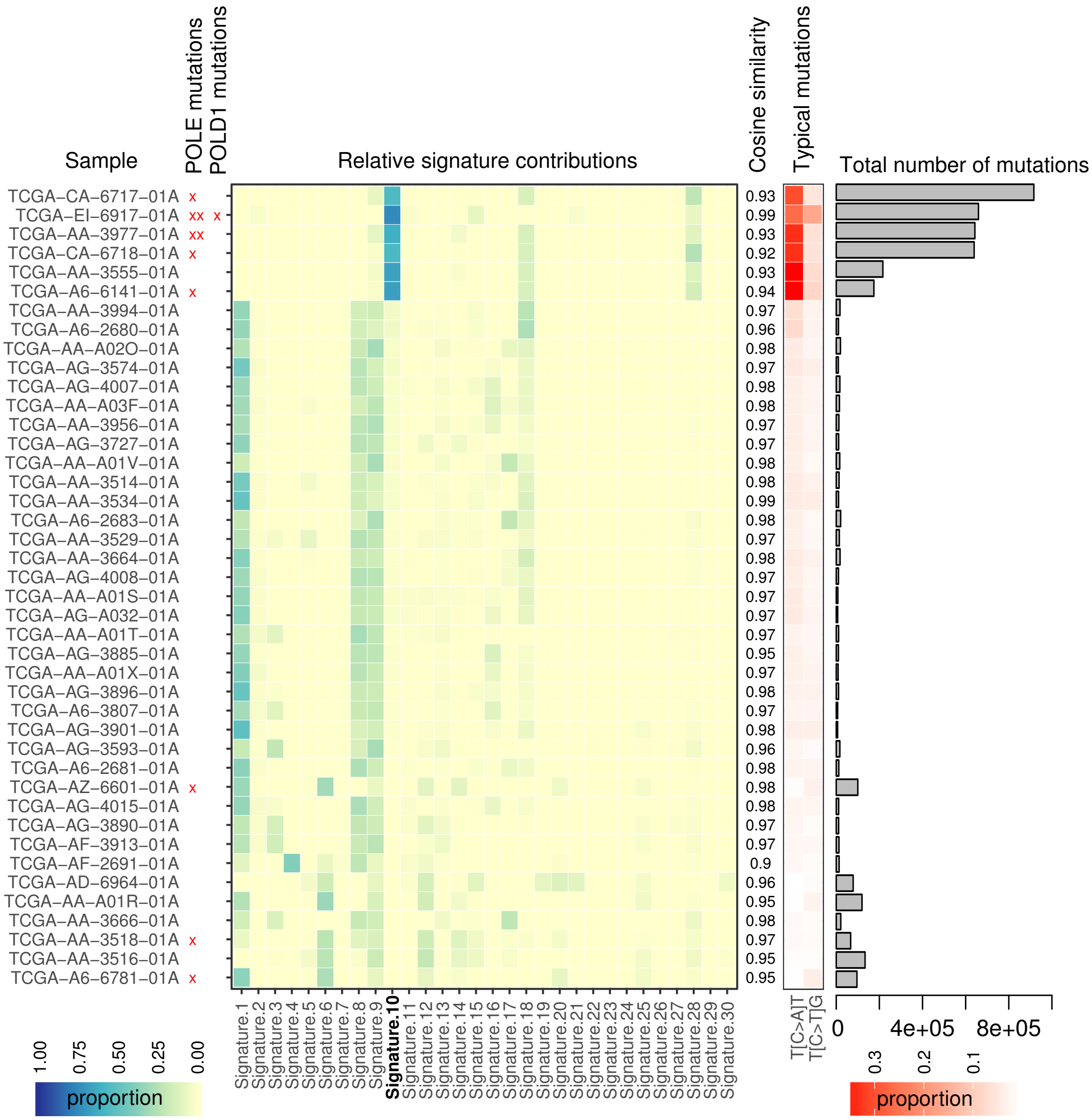

Figure S1: Detailed analysis of *POLE* mutation patterns in colon cancer samples. POLE and POLD1 mutations: nonsynonymous SNVs in *POLE* and *POLD1*. Relative signature contributions: proportion of mutations attributable to each of the 30 COSMIC signatures. Signature 10 has been found to capture the footprint of *POLE* mutations. Cosine similarity: the cosine similarity measures the similarity between the observed mutation vector and the mutation vector reconstructed with the COSMIC signatures (0 – orthogonal, 1 – identical). Typical mutations: Proportion of typical mutations seen in *POLE* and *POLD1* mutated tumors.

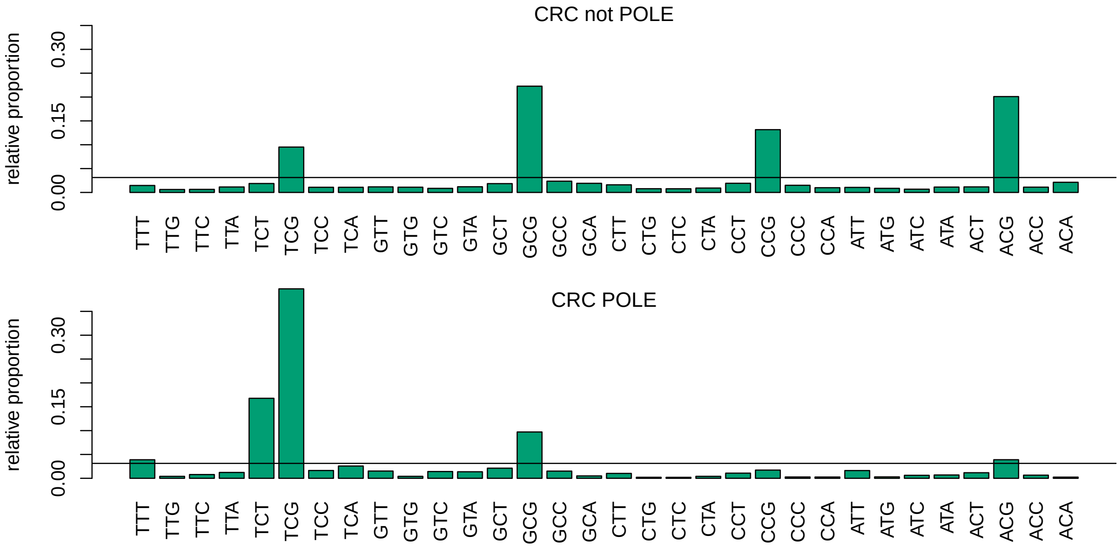

Figure S2: Proportion of observed mutations per nucleotide triplet in colon cancer samples. The proportions are adjusted to abundance of each triplet in the genome. Top panel: colon cancer samples with no POLE mutation pattern; bottom panel: colon cancer samples with POLE mutation pattern (the top six samples in fig. S1).

S2 Detailed forward model selection results

We use step by step forward model selection to identify important explanatory variables and to build up the final model. In S2.1, we show the improvement of the model in terms of deviance loss and McFadden's pseudo R^2 along the model selection procedure. To verify the robustness of the forward model selection procedure, we randomly sample another five datasets from the whole genome and run the model selection procedure. In S2.2, we show that the explanatory variables are selected in the same order for all the five datasets.

S2.1 Loss estimates for all models under consideration

Model	deviance loss	McFadden's pseudo R^2
Model 5 $+\beta^{\text{mut. type}}_{\text{phyloP},c} x_{\text{phyloP},i}$	0.0003322	0.2102
Model 5 $+\beta^{\text{mut. type}}_{\text{repl. timing},c} x_{\text{repl. timing},i}$	0.0003359	0.2124
Model 5 $+\beta^{\text{mut. type}}_{\text{element},c} x_{\text{element},i}$	0.0003391	0.2089
Model 5 $+\beta^{\text{mut. type}}_{\text{expr.},c} x_{\text{expr.},c,i}$	0.0003395	0.2100
Model 5 $+\beta^{\text{mut. type}}_{\text{GC cont.},c} x_{\text{GC cont.},i}$	0.0003400	0.2087
Model 5 $+\beta^{\text{mut. type}}_{\text{DNase1},c} x_{\text{DNase1},i}$	0.0003405	0.2076
Model 5 $+\beta^{\text{mut. type}}_{\text{repeat},c} x_{\text{repeat},i}$	0.0003406	0.2075
Model 5 $+\beta^{\text{mut. type}}_{\text{CGI},c} x_{\text{CGI},i}$	0.0003406	0.2074

Table S1: Deviance loss and McFadden's pseudo R^2 for each of the models tested in step 1 to obtain model 6.

Model	deviance loss	McFadden's pseudo R^2
Model 6 $+\beta^{\text{mut. type}}_{\text{repl. timing},c} x_{\text{repl. timing},i}$	0.0003291	0.2147
Model 6 $+\beta^{\text{mut. type}}_{\text{expr.},c} x_{\text{expr.},c,i}$	0.0003310	0.2132
Model 6 $+\beta^{\text{mut. type}}_{\text{GC cont},c} x_{\text{GC cont},i}$	0.0003315	0.2119
Model 6 $+\beta^{\text{mut. type}}_{\text{element},c} x_{\text{element},i}$	0.0003316	0.2117
Model 6 $+\beta^{\text{mut. type}}_{\text{DNase1},c} x_{\text{DNase1},i}$	0.0003319	0.2109
Model 6 $+\beta^{\text{mut. type}}_{\text{repeat},c} x_{\text{repeat},i}$	0.0003319	0.2108
Model 6 $+\beta^{\text{mut. type}}_{\text{CGI},c} x_{\text{CGI},i}$	0.0003320	0.2107

Table S2: Deviance loss and McFadden's pseudo R^2 for each of the models tested in step 2 to obtain model 7.

Model	deviance loss	McFadden's pseudo R^2
Model 7 $+\beta^{\text{mut. type}}_{\text{expr.},c} x_{\text{expr.},c,i}$	0.0003248	0.2258
Model 7 $+\beta^{\text{mut. type}}_{\text{element},c} x_{\text{element},i}$	0.0003279	0.2154
Model 7 $+\beta^{\text{mut. type}}_{\text{repeat},c} x_{\text{repeat},i}$	0.0003289	0.2154
Model 7 $+\beta^{\text{mut. type}}_{\text{CG cont.},c} x_{\text{CG cont.},i}$	0.0003289	0.2152
Model 7 $+\beta^{\text{mut. type}}_{\text{DNase1},c} x_{\text{DNase1},i}$	0.0003290	0.2150
Model 7 $+\beta^{\text{mut. type}}_{\text{CGI},c} x_{\text{CGI},i}$	0.0003291	0.2150

Table S3: Deviance loss and McFadden's pseudo R^2 for each of the models tested in step 3 to obtain model 8.

S2.2 Robustness of the model selection procedure

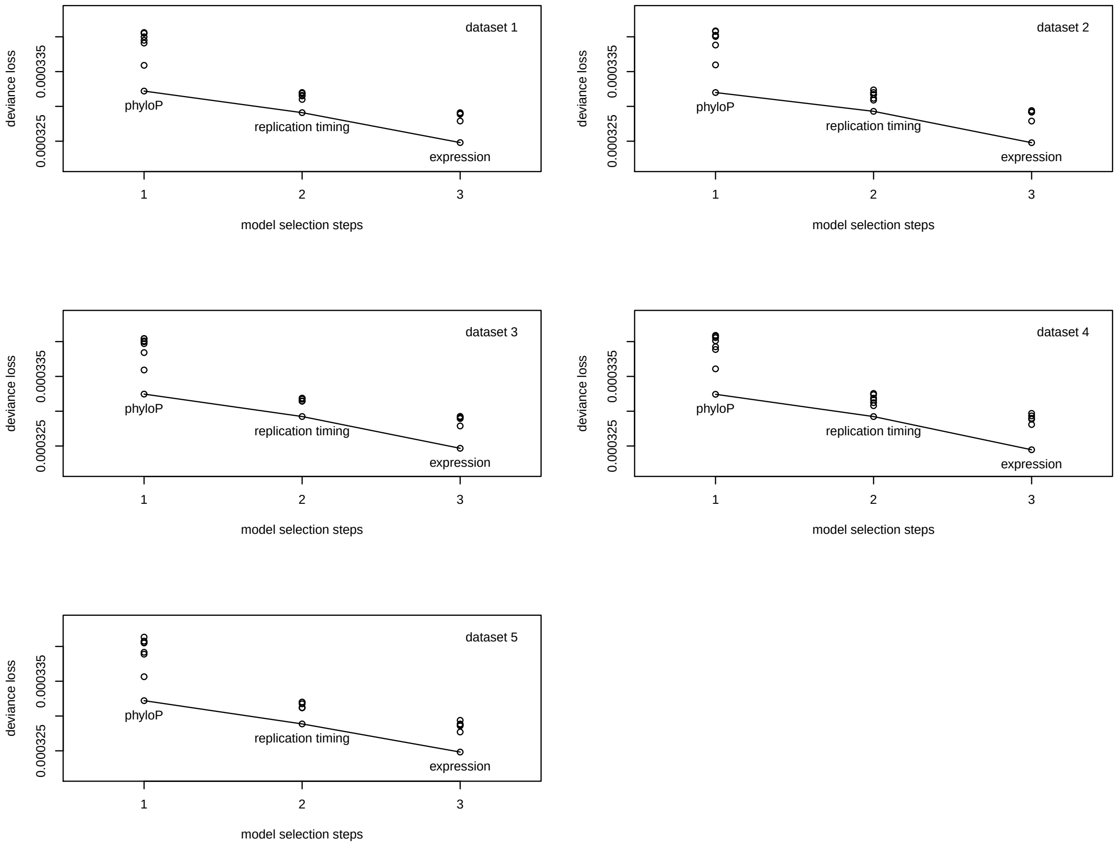

Figure S3: Model selection procedure on five datasets. In each step, explanatory variables are ranked according to the deviance loss of the corresponding models. Each dot represents one model. The best explanatory variable is added in the model step by step. For all the five datasets, the explanatory variables are added into the final model in the same order. PhyloP is the first one to be added, followed by replication timing in step 2 and expression level in step 3.

S3 Mutation patterns in two cancer genes

We compare the predicted number of mutations to the observed mutation patterns in two typical cancer genes, the oncogene *KRAS* (Fig. S4) and the tumor suppressor gene *TP53* (Fig. S5). Both of them are highly mutated in this dataset.

S3.1 The oncogene *KRAS*

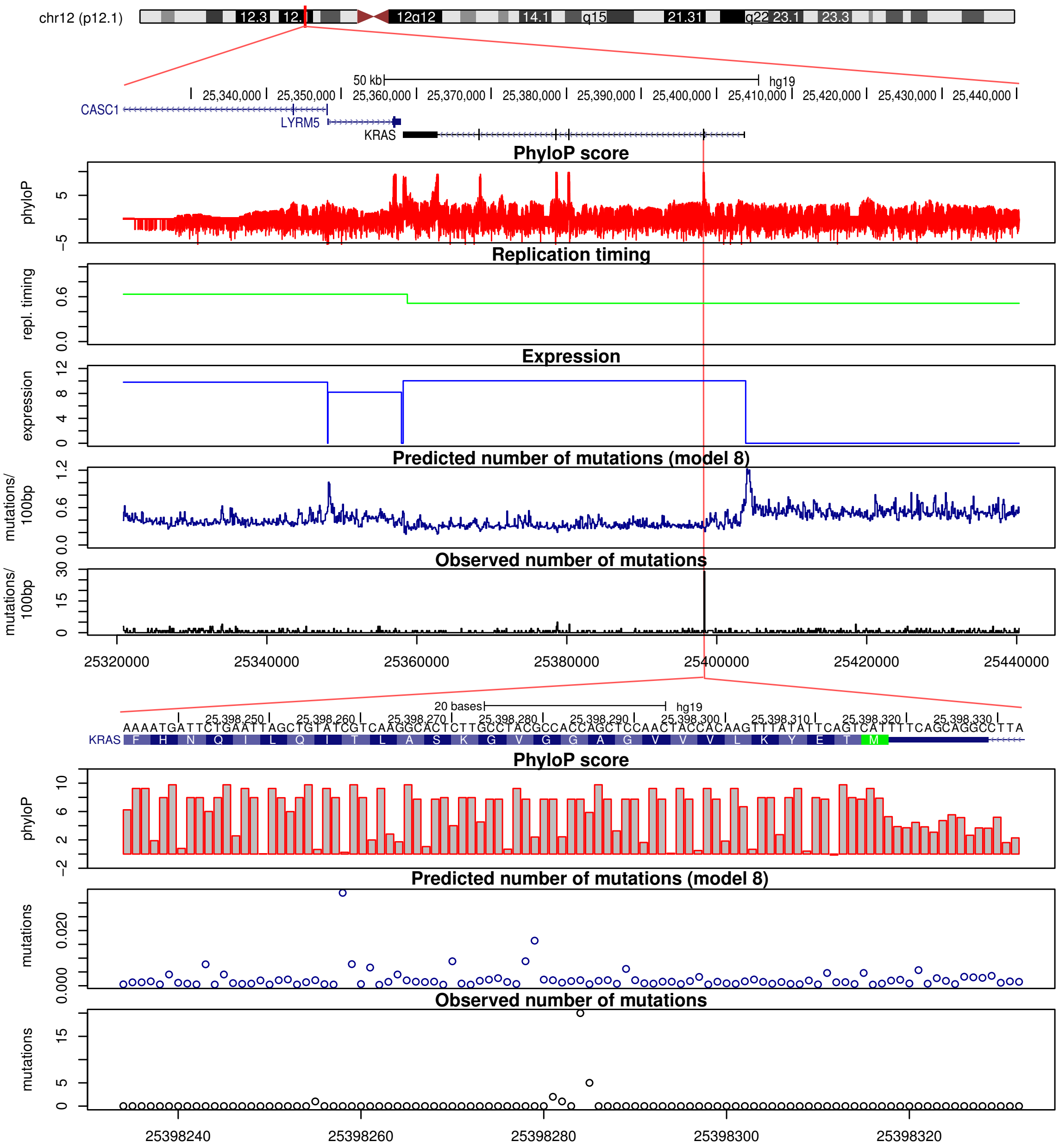

Figure S4: Explanatory variables, predictions and observed mutations in the oncogene *KRAS* and its genomic neighborhood. The chromosome ideogram and the gene annotations are obtained from the UCSC genome browser. To reduce the number of points of the figure, we plot a moving average over 100 bp for the phyloP score. The predicted number of mutations from the final model (model 8) and the observed number of mutations for all 505 samples in windows of 100 bp are plotted. Bottom: zoom into a region of exon 2 with a mutational hotspot. Here, the predicted number of mutations and the observed number of mutations for all 505 samples per position are plotted.

S3.2 The tumor suppressor gene *TP53*

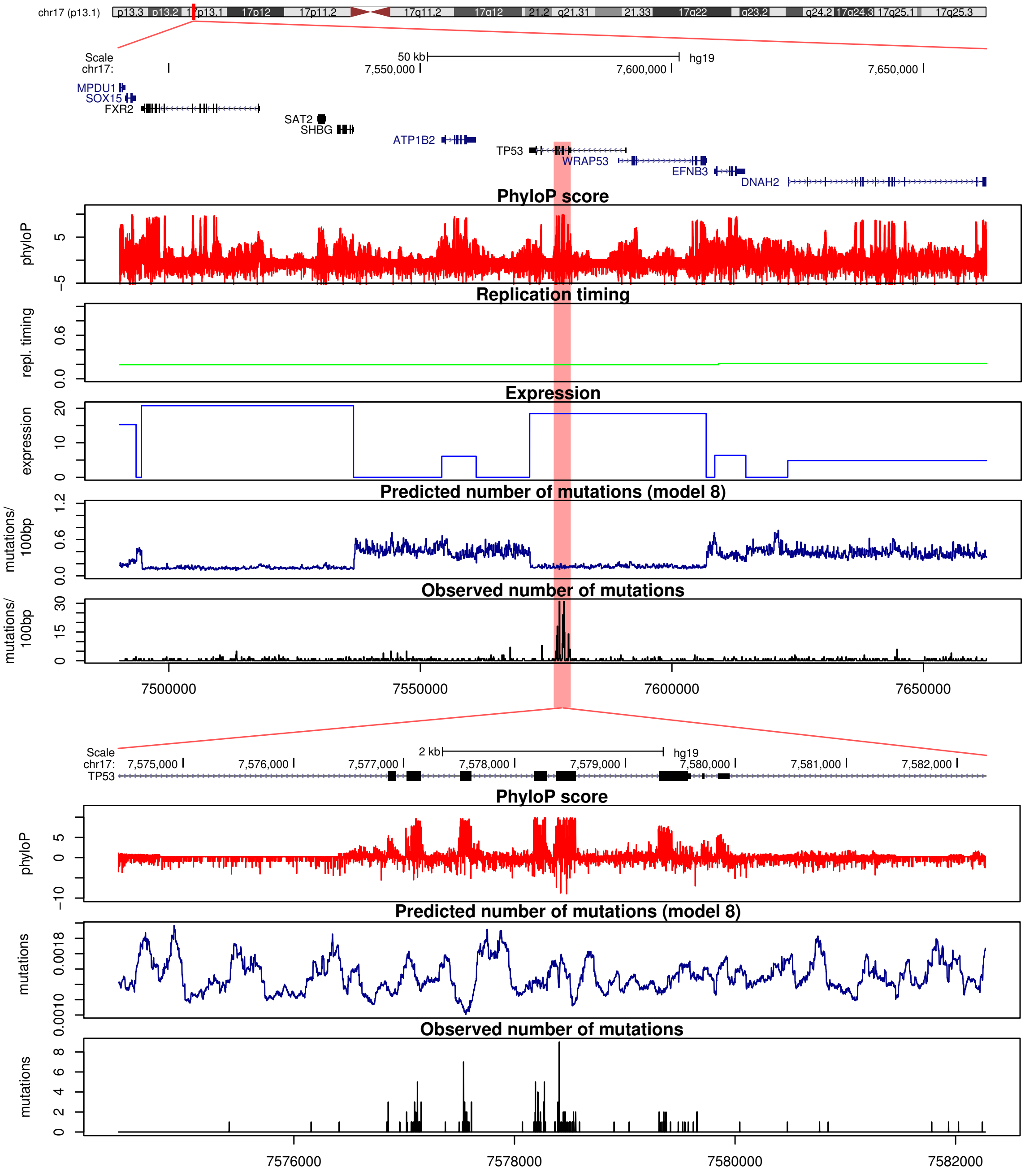

Figure S5: Explanatory variables, predictions and observed mutations in the tumor suppressor gene *TP53* and its genomic neighborhood. Annotations as in Fig. S4. Bottom: zoom into a region with multiple exons that harbor many mutations. Here, we plot a moving average over 100 bp for the predicted number of mutations.

References

L. B. Alexandrov, S. Nik-Zainal, D. C. Wedge, S. A. Aparicio, S. Behjati, A. V. Biankin, G. R. Bignell, N. Bolli, A. Borg, A.-L. Børresen-Dale, et al. Signatures of mutational processes in human cancer. *Nature*, 500:415–421, 2013.

L. B. Alexandrov, P. H. Jones, D. C. Wedge, J. E. Sale, P. J. Campbell, S. Nik-Zainal, and M. R. Stratton. Clock-like mutational processes in human somatic cells. *Nature Genetics*, 2015.

F. Blokzijl, R. Janssen, R. van Boxtel, and E. Cuppen. MutationalPatterns: comprehensive genome-wide analysis of mutational processes. *bioRxiv*, 2017. doi: 10.1101/071761. URL `https://www.biorxiv.org/content/early/2017/10/26/071761`.

Y. Fu, Z. Liu, S. Lou, J. Bedford, X. J. Mu, K. Y. Yip, E. Khurana, and M. Gerstein. FunSeq2: a framework for prioritizing noncoding regulatory variants in cancer. *Genome Biology*, 15(10):480, 2014. ISSN 1474-760X. doi: 10.1186/s13059-014-0480-5. URL `http://dx.doi.org/10.1186/s13059-014-0480-5`.
